# Construction and validation of a glycolysis-related lncRNA signature for prognosis prediction in Stomach Adenocarcinoma

**DOI:** 10.3389/fgene.2022.794621

**Published:** 2022-10-14

**Authors:** Tianyou Liao, Yan Lu, Wangji Li, Kang Wang, Yanxiang Zhang, Zhentao Luo, Yongle Ju, Manzhao Ouyang

**Affiliations:** ^1^ Department of Gastrointestinal Surgery, Shunde Hospital, Southern Medical University (The First People’s Hospital of Shunde Foshan), Foshan, Guangdong, China; ^2^ The Second School of Clinical Medicine, Southern Medical University, Guangzhou, Guangdong, China

**Keywords:** glycolysis, lncRNA, prognostic model, stomach adenocarcinoma, the cancer genome atlas

## Abstract

**Background:** Glycolysis is closely related to the occurrence and progression of gastric cancer (GC). Currently, there is no systematic study on using the glycolysis-related long non-coding RNA (lncRNA) as a model for predicting the survival time in patients with GC. Therefore, it was essential to develop a signature for predicting the survival based on glycolysis-related lncRNA in patients with GC.

**Materials and methods:** LncRNA expression profiles, containing 375 stomach adenocarcinoma (STAD) samples, were obtained from The Cancer Genome Atlas (TCGA) database. The co-expression network of lncRNA and glycolysis-related genes was used to identify the glycolysis-related lncRNAs. The Kaplan-Meier survival analysis and univariate Cox regression analysis were used to detect the glycolysis-related lncRNA with prognostic significance. Then, Bayesian Lasso-logistic and multivariate Cox regression analyses were performed to screen the glycolysis-related lncRNA with independent prognostic significance and to develop the risk model. Patients were assigned into the low- and high-risk cohorts according to their risk scores. A nomogram model was constructed based on clinical information and risk scores. Gene Set Enrichment Analysis (GSEA) was performed to visualize the functional and pathway enrichment analyses of the glycolysis-related lncRNA. Finally, the robustness of the results obtained was verified in an internal validation data set.

**Results:** Seven glycolysis-related lncRNAs (AL353804.1, AC010719.1, TNFRSF10A-AS1, AC005586.1, AL355574.1, AC009948.1, and AL161785.1) were obtained to construct a risk model for prognosis prediction in the STAD patients using Lasso regression and multivariate Cox regression analyses. The risk score was identified as an independent prognostic factor for the patients with STAD [HR = 1.315, 95% CI (1.056–1.130), *p* < 0.001] *via* multivariate Cox regression analysis. Receiver operating characteristic (ROC) curves were drawn and the area under curve (AUC) values of 1-, 3-, and 5-year overall survival (OS) were calculated to be 0.691, 0.717, and 0.723 respectively. Similar results were obtained in the validation data set. In addition, seven glycolysis-related lncRNAs were significantly enriched in the classical tumor processes and pathways including cell adhesion, positive regulation of vascular endothelial growth factor, leukocyte transendothelial migration, and JAK_STAT signaling pathway.

**Conclusion:** The prognostic prediction model constructed using seven glycolysis-related lncRNA could be used to predict the prognosis in patients with STAD, which might help clinicians in the clinical treatment for STAD.

## Introduction

Gastric cancer, also known as stomach cancer, is a common malignant tumor of the digestive system. Its morbidity and mortality were ranked fifth and fourth, respectively, among the global malignant tumors. More than half of the new cases were reported in developing countries with poor medical and health conditions. (Sung et al., 2021). Due to difficulties in early detection and rapid progress, the 5-year overall survival rate of gastric cancer (GC) is about 25–30%. (Siegel et al., 2016). In recent years, with advancements in the diagnosis and treatment of GC, the survival rate of early-stage GC has been significantly improved but its prognosis in the patients with advanced GC is still very poor (Shen et al., 2013; Song et al., 2017; Selim et al., 2019). This has become a prominent public health issue, threatening the health of people worldwide, especially in China. Due to the poor overall prognosis and large differences in the term of prognosis among GC patients (Costa et al., 2018), it is difficult to assess its prognosis in clinical practices. Therefore, the establishment of an evaluation system with good diagnosis and prognosis screening is particularly important for the diagnosis and treatment of GC. At present, in addition to the TNM (tumor node metastasis) staging system, which is commonly used in clinical practices, many studies (Eom et al., 2015; Tonello et al., 2021; Zhang and Yu, 2021) have proposed the construction of new classification and prognosis-related prediction models. However, most of these models are based on the general data and clinical pathological data of patients, thereby having a certain lag in the evaluation of tumor prognosis as compared to molecular indicators, such as genes. In addition, these factors are not conducive to the quantitative expression of prediction results, leading to the lack of accuracy and effectiveness. With the advent of the era of precision medicine and the rapid development of gene sequencing technologies, the tumor prognosis prediction models often incorporate molecular indicators, such as genes, long non-coding RNAs (lncRNAs), and miRNAs, to improve the performance of the prediction models.

Changes in cellular metabolism are closely related to the occurrence and development of multiple tumors, one of the most prominent changes is glycolysis (Ganapathy-Kanniappan, 2018; Liu et al., 2019; Orang et al., 2019). The energy acquisition of cells is mainly derived from glycometabolism. Normal cells mainly produce ATP through oxidative phosphorylation under aerobic conditions, while in hypoxic conditions, cells obtain ATP *via* glycolysis. In 1924, Warburg proposed the “*Warburg effect*”, demonstrating that the cancer tissues mainly obtain ATP through glycolysis; even in the presence of sufficient oxygen supply, they mainly use glycolysis to meet energy demand for their rapid growth. (Warburg, 1956). Aerobic glycolysis is the most basic metabolic change in the process of tumor malignancy, this phenomenon is widely present in various tumors (Chen et al., 2019; Weng et al., 2020; Zhang et al., 2020). Glycolysis promotes the proliferation of tumor cells, enhances invasiveness, and participates in tumor resistance. (Gatenby and Gillies, 2007; Akram, 2013). This phenomenon has also been found in GC (Liu et al., 2019) and indicates that aerobic glycolysis can lead to tumor progression and poor prognosis. (Gatenby and Gillies, 2004). Glycolysis can not only quickly supply energy and change the tumor microenvironment but also provides precursors or intermediates, such as nucleic acids, required for cell synthesis (Yu et al., 2017; Hofmann, 2018), which is considered an optimized way for the oncocytic cells to respond to cellular stress. This makes the enzymes and signaling pathways related to glycometabolism potential targets for the treatment of tumors. (Gatenby and Gillies, 2007).

In the past few decades, people have made great advancements in studying the molecular mechanisms of GC (Carlomagno et al., 2017; Ye et al., 2020). However, there is still a lack of molecular markers in the treatment and prognosis assessment of GC. LncRNA, a type of RNA with ≥200 nucleotides length, has no functions on encoding the proteins. (Cabili et al., 2011). Recent studies have shown that the lncRNAs are widely distributed in the genome and participate in the regulation of chromatin modification, gene transcription, and other important physiological processes, which are closely related to the initiation and progression of the tumor, and can be used as a potential biomarker for the diagnosis and prognosis assessment of tumor (Ponting et al., 2009; Gómez-Maldonado et al., 2015). However, the mechanisms of action of glycolysis-related lncRNA in GC patients are still unclear. Furthermore, the potential values of glycolysis-related lncRNAs in the diagnosis, treatment and prognosis evaluation of GC are still needed to be explored. Therefore, a glycolysis-related lncRNA signature and a nomogram were constructed in this study to provide clinicians with a quantitative method for predicting the survival in GC patients. This might help clinicians to make accurate and personalized clinical decisions and prognostic assessments for the patients with GC.

## Materials and methods

### Sample data sets and data pre-processing

From the Cancer Genome Atlas (TCGA) database (https://portal.gdc.cancer.gov/), the RNA-seq data of 375 cases and clinical data of 443 cases in stomach adenocarcinoma (STAD) were obtained. In the TCGA official website, “transcriptome profiling” was selected in Data Category, “Gene Expression Quantification” was selected in Data Type, “RNA-Seq” was selected in Experimental Strategy, and “illumine” was selected in Platform. The inclusion criteria of this study were: 1) Pathological diagnosis was stomach adenocarcinoma; and 2) Patients had complete sequencing data and clinical information (gender, age, distant metastasis, lymphatic metastasis, primary tumor, TNM Stage, and survival status). In addition, when the clinical information of the patients was screened, those with a follow-up period of ≤30 days were excluded. After screening, a total of 337 STAD samples, having both the lncRNA expression and prognostic data, were included for the construction and verification of the prognostic model; among which, 296 STAD samples, having complete clinical and prognostic data, were selected to perform the univariate and multivariate Cox regression analysis.

### Screening of lncRNA and identification of glycolysis-related LncRNA

First, the gene name data was used in the HUGO Gene Nomenclature Committee (HGNC, https://www.genenames.org) database to identify and isolate the lncRNA data from all the RNA-seq data sets to obtain lncRNA expression profiles. The total lncRNA expression data was normalized using log2 transformation. The glycolysis-related gene list was obtained from the Molecular Signatures Database (MSigDB) (https://www.gsea-msigdb.org/GSEA/msigdb). “Glycolysis” was used as a keyword to search for the glycolysis-related gene set, “REACTOME_GLYCOLYSIS”, which was then downloaded. Then, the Pearson correlation analysis was performed to calculate the correlations between lncRNAs and glycolysis-related genes. The lncRNAs, having correlation coefficient |R (Siegel et al., 2016)| >0.3 and *p*-value < 0.001, were considered glycolysis-related lncRNAs. Subsequently, the co-expression network was drawn using Cytoscape (Version 3.7.1) to visualize the correlations.

### Development and analysis of prognostic prediction model

First, a univariate Cox regression analysis was performed to evaluate the prognostic value of the identified glycolysis-related lncRNA. In univariate Cox regression analysis, all the glycolysis-related lncRNAs with a *p*-value < 0.05 were considered as related to the prognosis of STAD patients. The glycolysis-related lncRNAs with prognostic significance analysis were included to perform Lasso regression analysis. Then, the lncRNAs, which were obtained *via* Lasso regression analysis, were incorporated into a multivariate Cox regression model for the construction of a risk scoring model. The risk score for each patient was calculated using the following equation: Risk score = Coef lncRNA 1) × Expr lncRNA 1) + Coef lncRNA 2) × Expr lncRNA 2) + ... + Coef lncRNA (n) × Expr lncRNA (n). Coef value was the regression coefficient obtained through multivariate Cox regression analysis, and Expr lncRNA (n) was the expression of lncRNA (n). The patients were assigned into high- and low-risk cohorts according to the median value of risk score. Log-Rank (Mantel-Cox) test was used to compare the survival differences between the two cohorts. The survival program package in R 3.6.3 software was used to draw the survival curve of the model prognosis and compare the differences in survival between the two cohorts. SurvivalROC package in R software was used to draw the receiver operating characteristic (ROC) curve and the area under curve (AUC) value was calculated to evaluate the sensitivity and specificity of the prognostic model.

### Construction and analysis of nomogram graph

A nomogram graph was constructed to predict the survival time of patients with STAD. In order to verify the accuracy of the nomogram, the index of concordance (C-index) was calculated and calibration curves were drawn. The nomogram can provide a quantitative prediction method for clinicians and decision-makers in health-related departments. Therefore, a nomogram was constructed based on risk scores and clinicopathological information. In addition, the calibration curves for 1-, 3- and 5-year survival time were drawn concurrently. The closer the calibration curve is to the standard curve, the better the prediction model’s performance. Then, the clinical data, including demographic data, pathological TNM stage, pathological tumor, node, metastasis stage and patient’s risk score, were merged and those lacking accurate clinical data were deleted. In order to verify and compare the efficacy of the constructed prognostic signature with other clinical prognostic factors, a multivariate ROC curve was drawn. Furthermore, the AUCs of multiple factors, including age, gender, TNM stage, pathological grade and risk score, were calculated.

### Gene Set Enrichment Analysis (GSEA) and correlation analysis

Although the usability of prognostic signature was tested for survival predictions, it was unclear how the function of glycolysis-related lncRNA would work. Therefore, the GSEA analysis, including Gene Ontology (GO) analysis and Kyoto Encyclopedia of Genes and Genomes (KEGG) pathways analysis, was performed to explore the functional enrichment of glycolysis-related lncRNAs. The top five enrichment analysis results of GO and KEGG were presented. All the RNA-seq data of patients with STAD were also assigned into the low- and high-risk cohorts according to the median value of risk score. In the first, the previously obtained risk score data and lncRNA expression data were integrated and converted into “cls” and “gct” file formats. Subsequently, these data files were imported into GSEA software (version 4.0.3) to explore significant differences in the functions of glycolysis-related lncRNA between the low- and high-risk groups. Furthermore, correlations between risk level and clinicopathological characteristics were calculated using Pearson’s correlation coefficient analysis, where coefficient >0 indicated a positive correlation and coefficient <0 indicated a negative correlation.

### Model verification

A total of 170 samples were randomly selected from 337 STAD samples having both the RNA-seq data of lncRNA and prognostic data to form an internal cross-validation data set, which was used to verify the constructed prediction model and evaluate its robustness. The Kaplan-Meier plots between the high- and low-risk groups, ROC curve of multiple factors including clinical information and risk score, and univariate and multivariable Cox regression analysis were performed using similar methods as described in above-mentioned section.

In addition, we performed 5-fold cross-validation to make the verification results more reliable. 337 STAD samples were divided into five cohorts where 80% of the data was used as training data and remaining 20% was used as a validation set, and then we repeat this five times so that every cohort serve as a validation set. The accuracy measures (AUC, confusion matrix, sensitivity, specificity) was reported.

This process was performed to identify consistency in the conclusions of training and verification cohorts to evaluate the robustness and reliability of the risk prognosis model.

### Statistical analyses

Kaplan-Meier method was used to draw survival curve and Log-rank test was used to compare the survival curve between high-risk and low-risk cohorts. Cox regression analysis and Lasso regression analysis were performed to screen the glycolysis-related lncRNAs and clinical information that have a prognostic impact for the patients with STAD. The ROC curves were drawn to evaluate the performance of the prediction model. Univariate and multivariate Cox regression analyses were performed to recognize the predictors in clinical variables and risk scores, and then, the usability of the risk model as an independent prognostic indicator was assessed. Strawberry PERL (version 5.30.2.1) was used to process the data. All the statistical analyses in this study were performed using R software (version 4.0.2). The statistical test was two-sided, and *p* < 0.05 was considered statistically significant. The prediction with AUC >0.60 was considered to be an acceptable prediction, while the AUC >0.75 was considered to have a good predictive value.

## Results

### Co-expression network construction

The detailed flow chart for the prognostic predictive model construction in this study was shown in Supplementary Figure S1. A total of 14,142 lncRNAs in the TCGA-STAD data sets were extracted from the TCGA database and 72 glycolysis-related genes were extracted from the GSEA-MSigDB database, among which, 70 genes were expressed in STAD (Supplementary Table S1). The co-expression network of glycolysis-related lncRNA was constructed to identify lncRNA related to glycolysis. Finally, a total of 870 lncRNAs were identified as glycolysis-related lncRNAs (|R (Siegel et al., 2016)|>0.3, and *p* < 0.001).

### Construction of glycolysis-related lncRNA signature

**FIGURE 1:**
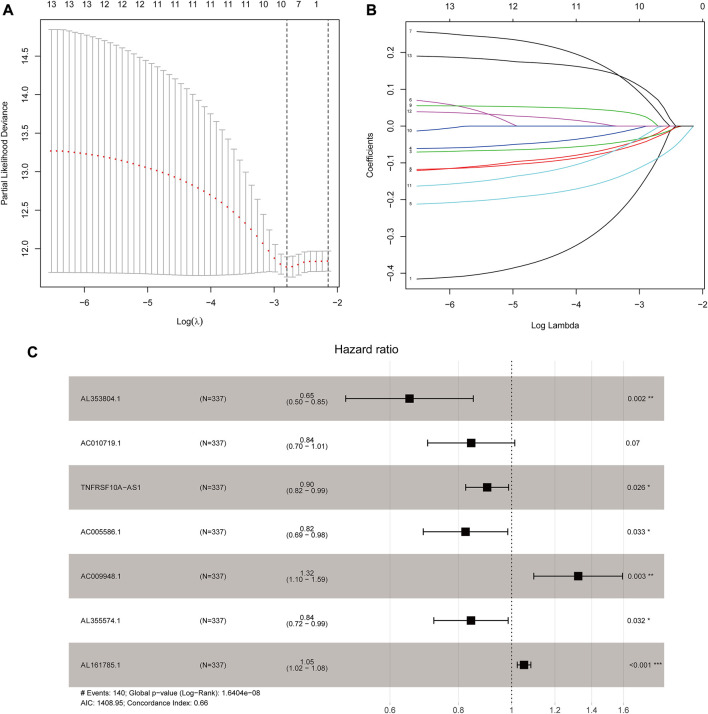
Development of the seven glycolysis-related lncRNAs signature. **(A)** Lasso coefficient values of nine glycolysis-related lncRNAs in stomach adenocarcinoma (STAD). The vertical dashed lines are at the optimal log(lambda) value. **(B)** Variables going to zero as we increase the penalty (lambda) in the objective function of the Lasso. **(C)** Results of the multivariate Cox proportional hazard model based on the nine variables; seven lncRNAs were screened to construct the signature.

**FIGURE 2:**
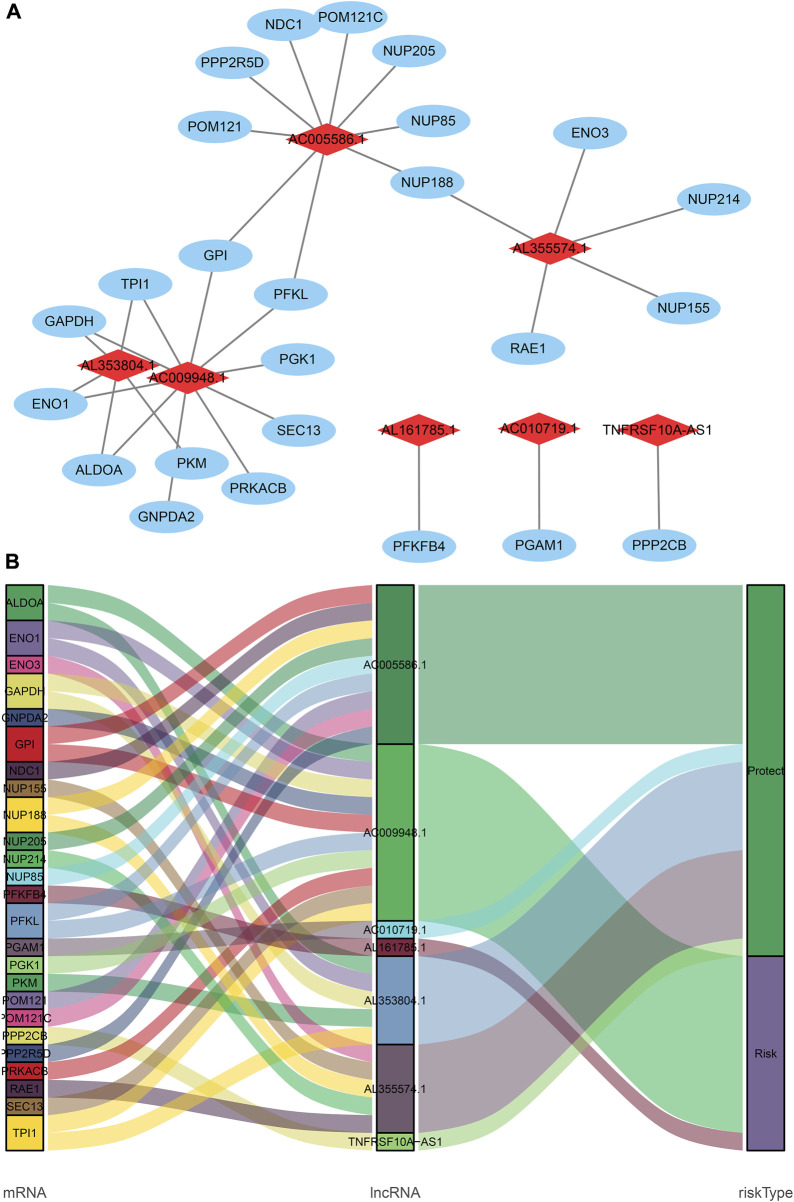
The coexpression network and Sankey diagram of prognostic glycolysis-related lncRNAs. **(A)** The coexpression network between prognostic glycolysis-related lncRNAs and glycolysis-related genes in STAD. Red diamond nodes represent prognostic glycolysis-related lncRNAs, and the sky-blue oval nodes represent glycolysis-related genes. The coexpression network was visualized using Cytoscape 3.7.1 software. **(B)** Sankey diagram showed the association between prognostic glycolysis-related lncRNAs, glycolysis--related genes, and risk types.

**FIGURE 3:**
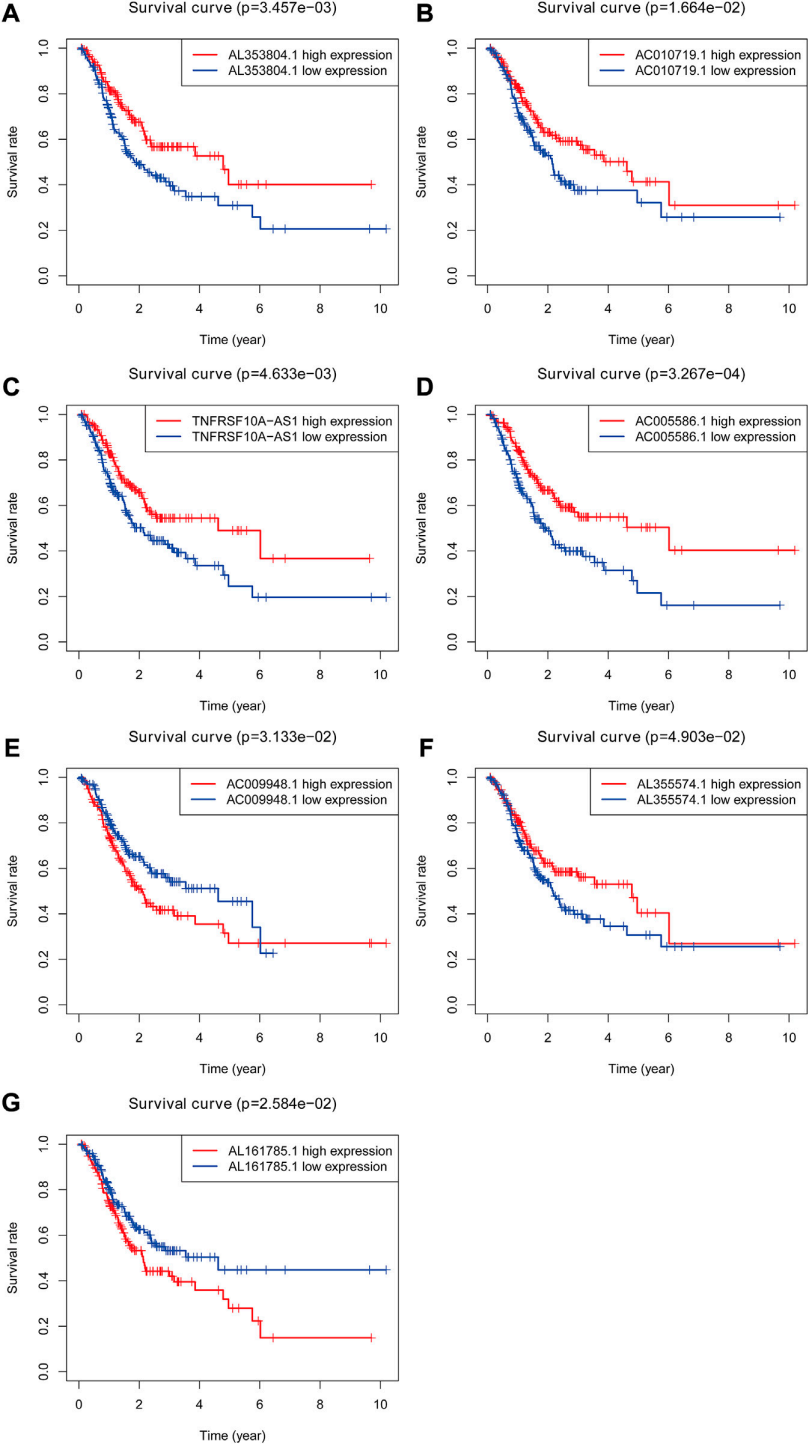
The Kaplan-Meier Survival curve of seven prognostic glycolysis-related lncRNAs. Two glycolysis-related lncRNAs (AC009948.1 and AL161785.1) were independent unfavorable factors. Five lncRNAs (AL353804.1, AC010719.1, TNFRSF10A-AS1, AC005586.1 and AL355574.1) were independent beneficial factors for STAD.

Based on 870 glycolysis-related lncRNAs, univariate Cox regression analysis was used to screen the glycolysis-related lncRNAs having prognostic significance in the 337 cases of TCGA-STAD data set. The univariate Cox regression analysis showed that there were 13 lncRNAs (AL353804.1, AC010719.1, TNFRSF10A-AS1, MAPKAPK5-AS1, AC005586.1, SREBF2-AS1, AC009948.1, AL355574.1, AL161785.1, AC084033.3. AP003392.1, AC037198.1, and AP001528.2), having significant prognostic value for STAD patients (*p* < 0.05, Supplementary Table S2). Subsequently, the Lasso regression analysis was used to avoid overfitting. After performing the Lasso regression, nine glycolysis-related lncRNAs were identified (Figure 1; Supplementary Table S3). Then, these lncRNAs were subjected to multivariate Cox regression analysis and a prognostic signature with seven glycolysis-related lncRNAs was developed. Among them, five lncRNAs (AL353804.1, AC010719.1, TNFRSF10A-AS1, AC005586.1, and AL355574.1) were identified as favorable prognostic factors, while the remaining two lncRNAs (AC009948.1 and AL161785.1) were presented as poor prognostic factors (Table 1). Subsequently, these seven lncRNAs were used to develop a glycolysis-related lncRNAs signature. Risk score = (-0.42868* AL353804.1) + (-0.16952* AC010719.1) + (-0.10245* TNFRSF10A-AS1) + (-0.19337* AC005586.1) + (0.279,944* AC009948.1) + (-0.17044* AL355574.1) + (0.052446* AL161785.1). In addition, a co-expression network of prognostic-related glycolytic lncRNAs was constructed (Figure 2). Simultaneously, a combined Sankey diagram was drawn based on 25 glycolysis-related genes, seven glycolysis-related lncRNAs and their risk conditions (Figure 3).

**TABLE 1:** Multivariate Cox results of glycolysis-related lncRNAs in STAD.

lncRNA	Coefficient	HR	HR.95L	HR.95H	*p* Value
AL353804.1	−0.429	0.651	0.498	0.852	0.002
AC010719.1	−0.170	0.844	0.703	1.014	0.070
TNFRSF10A-AS1	−0.102	0.903	0.825	0.988	0.026
AC005586.1	−0.193	0.824	0.690	0.984	0.033
AC009948.1	0.280	1.323	1.098	1.594	0.003
AL355574.1	−0.170	0.843	0.721	0.986	0.032
AL161785.1	0.052	1.054	1.024	1.084	<0.001

### Glycolysis-related lncRNA signature for prognosis prediction in STAD

**FIGURE 4:**
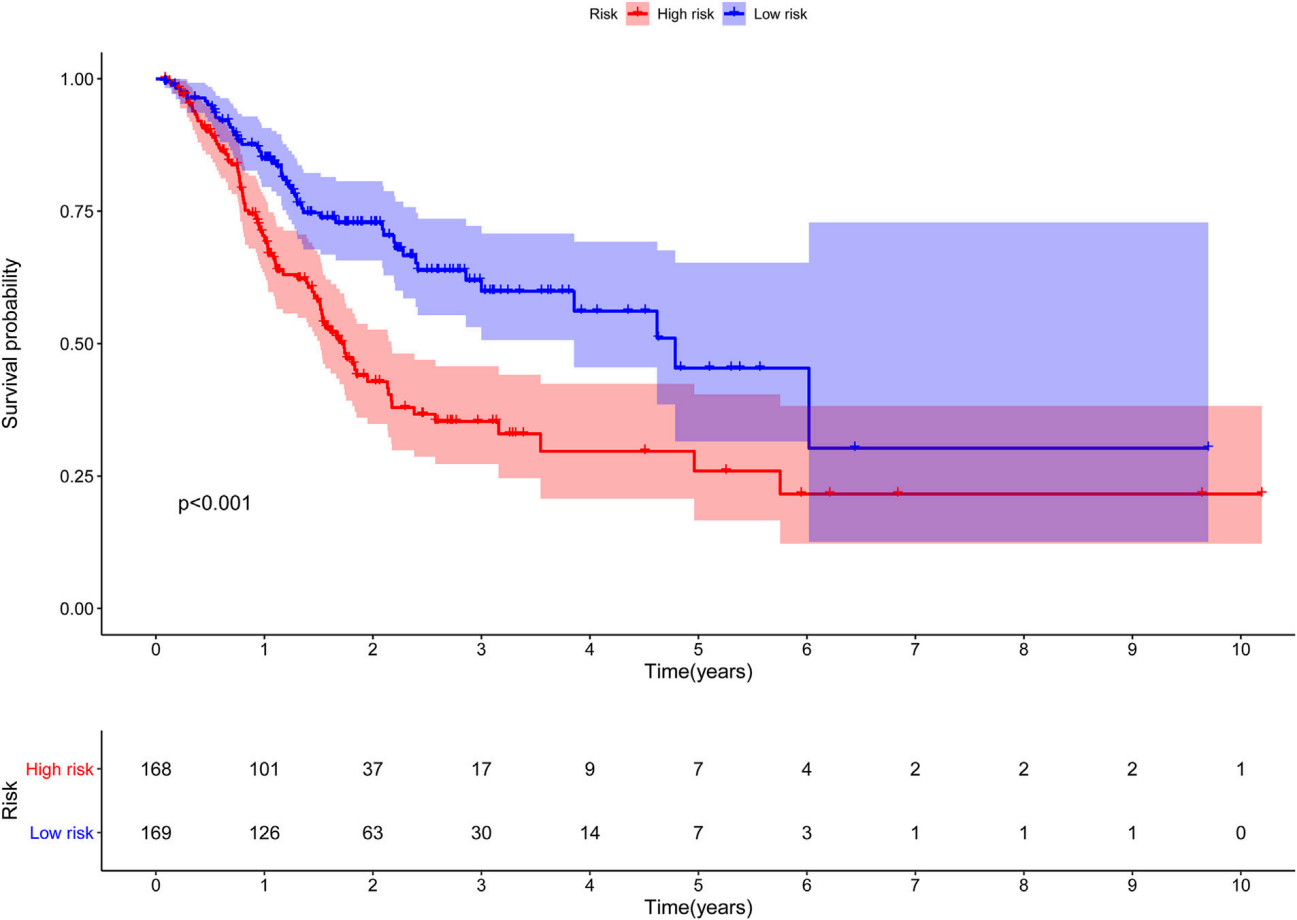
The Kaplan-Meier survival curve of risk score based on seven glycolysis-related lncRNAs.

**FIGURE 5:**
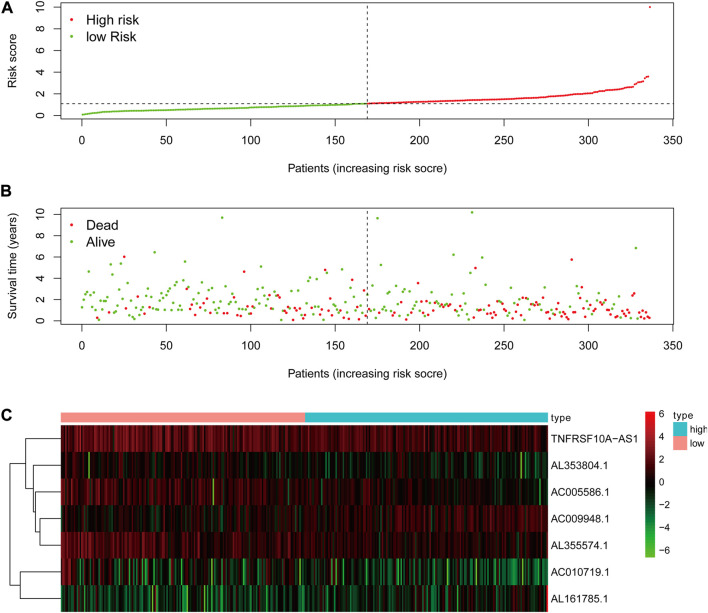
The analysis of glycolysis-related lncRNA signature for patients in STAD. **(A)** The risk score between the high-risk group and low-risk group. **(B)** The survival status of the high-risk group and the low-risk group in STAD patients. **(C)** Heat map of seven glycolysis-related lncRNAs’ expression. The color from green to red reveals a rising tendency from low to high expression levels.

The risk score of the glycolysis-related lncRNA risk model was calculated using the above risk score formula (Table 2). Survival program package in R software was used to analyze survival differences based on the patient’s risk score and the risk score curve, survival status map, and heat map based on the expression level of seven lncRNAs were drawn using the R programming language. Kaplan-Meier plot showed that the risk score was significantly correlated with overall survival (OS) of patients with STAD. As compared to the low-risk cohort, the OS of the high-risk cohort was significantly shorter (*p* < 0.001, Log-rank test) (Figure 4). With the increase in risk score, the number of patients’ deaths in the high-risk cohort was significantly higher than that in the low-risk cohort, indicating the poor OS of STAD patients in the high-risk cohort (Figure 5). The above results showed that the risk score was significantly correlated with the OS of patients with STAD and exhibited a significant impact on the prognosis in STAD. It was suggested that the risk signature might better predict the survival and prognosis in STAD.

**TABLE 2:** Multivariate Cox regression based on clinical characteristics and risk scores in STAD.

lncRNA	B	SE	Z	HR	HR.95L	HR.95H	*p* Value
Age	0.027	0.010	2.753	1.027	1.008	1.047	0.006
Gender	0.277	0.201	1.374	1.319	0.889	1.956	0.169
Stage	0.256	0.211	1.215	1.292	0.855	1.952	0.224
T	0.088	0.154	0.573	1.092	0.808	1.476	0.567
M	0.243	0.429	0.566	1.275	0.550	2.955	0.571
N	0.147	0.117	1.257	1.159	0.921	1.458	0.209
Risk Score	0.088	0.018	4.905	1.092	1.054	1.131	<0.001

### Clinical value and significance of the glycolysis-related lncRNA signatures

**FIGURE 6:**
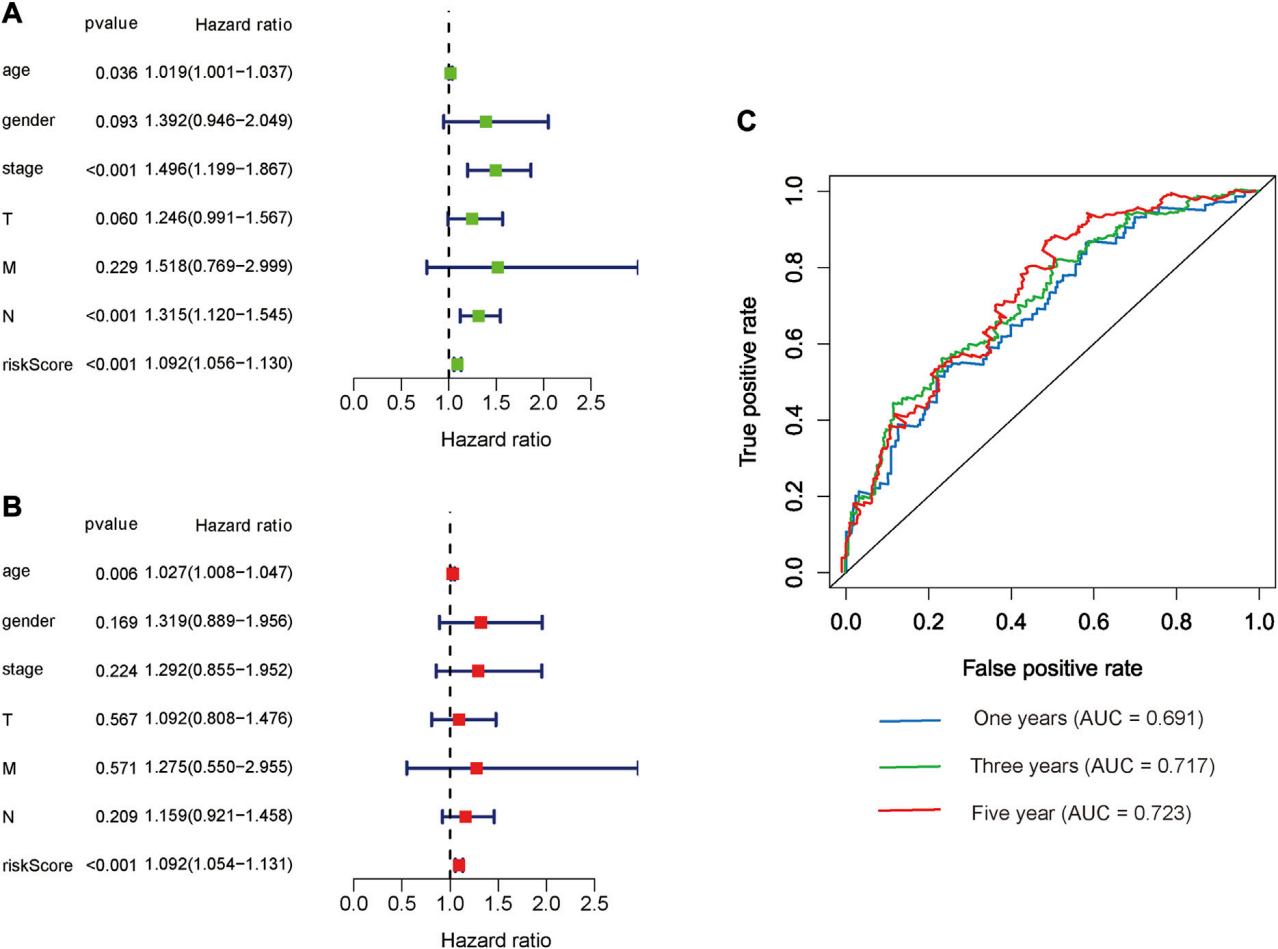
Prognostic indicators based on glycolysis-related lncRNAs showed great predictive performance. The forest plots for univariate **(A)** and multivariate **(B)** Cox regression analysis in STAD. **(C)** The areas under the ROC curve about 1-year, 3-year, and 5-year OS.

**FIGURE 7:**
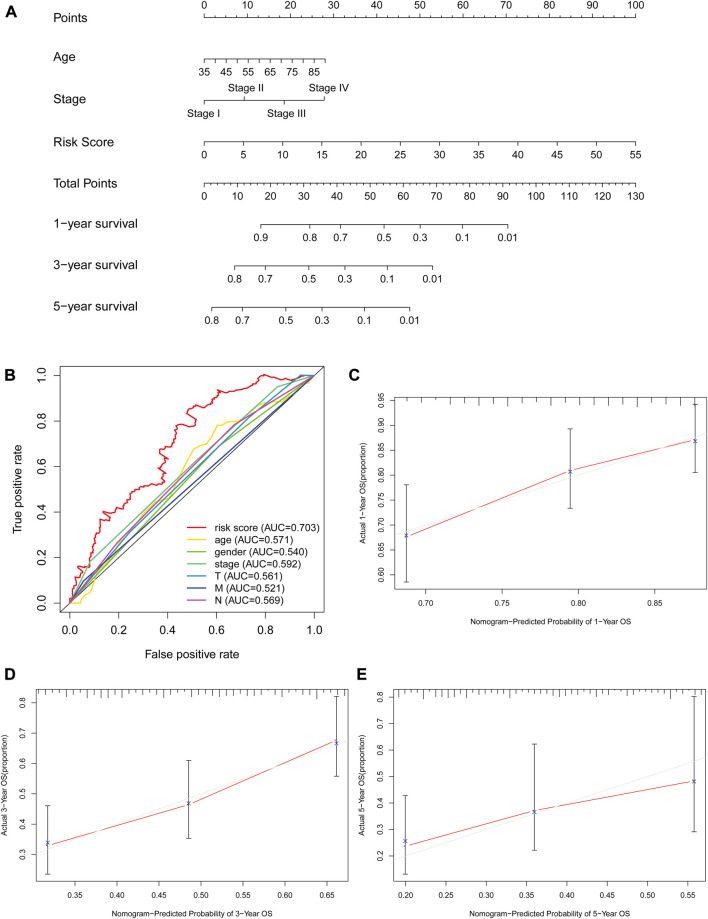
The evaluation of prognostic models based on seven glycolysis-related lncRNAs. **(A)** The nomogram of 1-, 3- and 5-year OS based on risk score, age, and TNM stage. **(B)** The ROC curves analysis based on risk score and the clinicopathologic parameters; **(C–E)** The calibration plots of 1-, 3- and 5-year OS for evaluating the concordance between the predicted and the standard OS for the prognosis model. The closer the calibration curve (red line) is to the standard curve (grey line), the better the prediction model’s performance.

The risk score, lymph node status and pathological TNM stage were prognostic indicators identified by univariate Cox regression analysis. The Cox regression result of risk score are as follow: [HR = 1.315, 95% CI (1.056–1.130), *p* < 0.001], (Figure 6A). When the influence of other factors (gender, age, and tumor stage) was controlled and eliminated, the risk score was still identified as an independent prognostic indicator according to multivariate Cox regression analysis [HR = 1.092, 95% CI (1.054–1.131)), *p* < 0.001], (Table 2; Figure 6B). Subsequently, the survivalROC program package was used to draw the ROC curve of the risk model and evaluate its sensitivity and specificity. The results showed that the calculated AUC values of the prognostic signatures for 1-, 3-, and 5-year survival time were 0.691, 0.717, and 0.723, respectively (Figure 6C). Furthermore, the risk score, TNM stage, and age were used to construct a nomogram. As shown in Figure 7A, the risk score was the most important contributing factor to the 1-, 3-, and 5-year OS in STAD. The calculated C-index of the prediction model was 0.651 (95% CI: 1.056–1.135). The 5-year AUC of risk score was 0.703, which was higher than age (AUC = 0.571), gender (AUC = 0.540), TNM stage (AUC = 0.592), tumor stage (AUC = 0.561), node stage (AUC = 0.569), and distal metastasis stage (AUC = 0.521) (Figure 7B). The correlation analyses found that the level of risk score was not significantly correlated with age, gender, TNM staging, etc. (Table 3).

**TABLE 3:** Clinical influences of risk score signature for TCGA-STAD data.

Clinical	Group	*n*	Risk score		*t*	*P*
			Mean	SD		
Age	>60	194	1.35	3.613	0.354	0.724
	≤60	102	1.256	0.649		
Gender	0	110	1.066	0.636	-1.444	0.15
	1	186	1.466	3.681		
Stage	I-II	135	1.454	4.317	0.669	0.505
	III-IV	161	1.203	0.615		
T	1-2	74	1.781	5.806	0.915	0.363
	3-4	222	1.163	0.602		
M	0	277	1.314	3.042	-0.197	0.844
	1	19	1.362	0.688		
N	0	90	1.013	0.586	-1.734	0.084
	1-3	206	1.45	3.506		

### GSEA analysis

**FIGURE 8:**
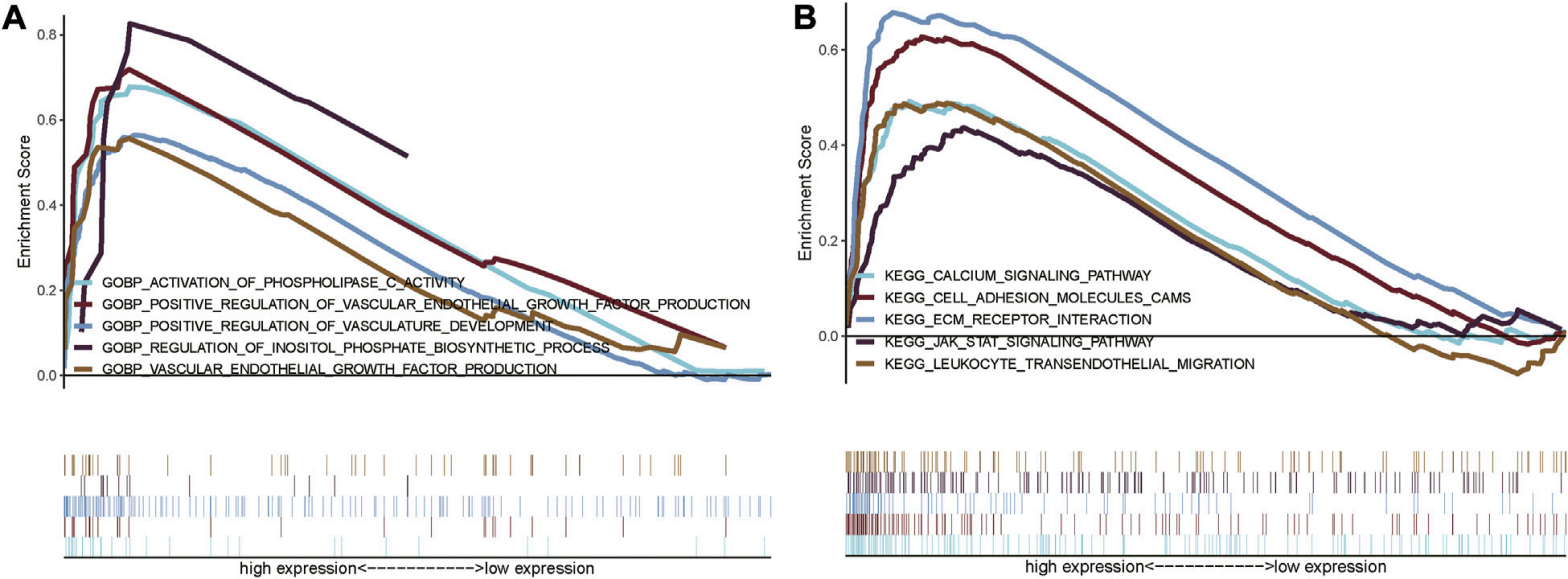
Gene set enrichment analysis showing the GO and KEGG enrichment between the high-risk and low-risk group. **(A)** GSEA for GO enrichment analysis; **(B)** GSEA for KEGG enrichment analysis.

In order to explore the functional differences in the glycolysis-related lncRNAs between the high- and low-risk groups, GO and KEGG enrichment analyses were performed using GSEA version 4.0.3, which are shown in Figures 8A,B. A total of 3009 GO entries and 95 KEGG pathways were obtained. In GO analysis, the enrichment results were mainly concentrated on the regulation of inositol phosphate biosynthetic process and vasculature development, activation of phospholipase C activity, positive regulation of vascular endothelial growth factor production, vascular endothelial growth factor production, etc. (Figure 8A). KEGG pathways analysis showed that the glycolysis-related lncRNAs were mainly involved in cell adhesion molecules (CAMs), extracellular matrix (ECM) receptor interaction, calcium signaling pathway, leukocyte transendothelial migration, and JAK_STAT signaling pathway (Figure 8B). In addition, it was also found that these enrichment results were related to important biological processes and functional pathways in the initiation and progression of the tumor. For instance, the regulation of vasculature development and vascular endothelial growth factor production, cell adhesion, leukocyte transendothelial migration, and JAK_STAT signaling pathway were closely related to the proliferation, invasion, and metastasis of tumor.

### Verification in the internal verification set

**FIGURE 9:**
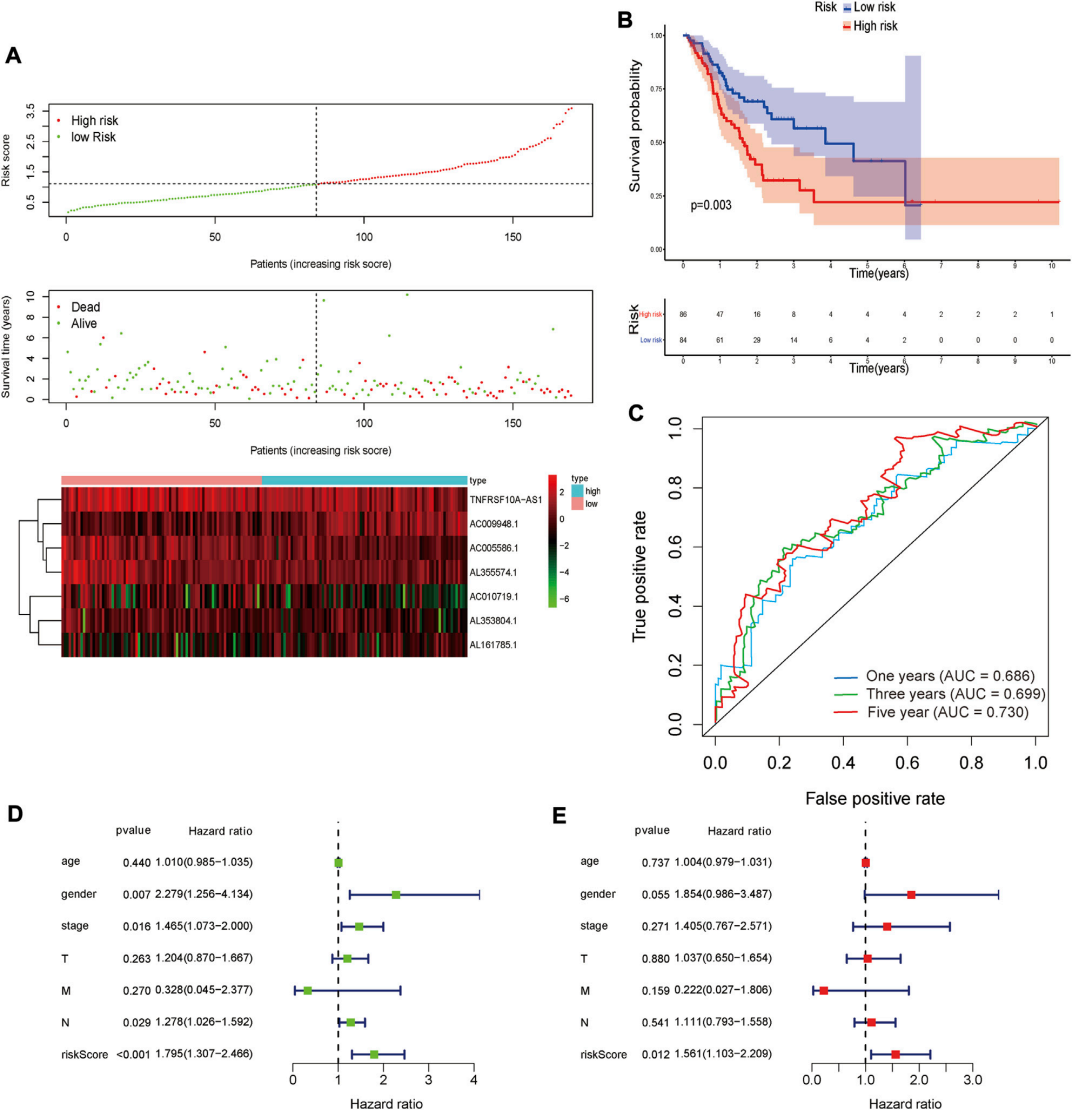
The result of validation cohort based on seven glycolysis-related lncRNAs in STAD. **(A)** The risk score, survival status and heat map of seven glycolysis-related lncRNAs between the high-risk and the low-risk group in validation cohort. **(B)** Kaplan-Meier survival curve of risk score in validation cohort. **(C)** The areas under the ROC curve about 1-, 3-, and 5-year in validation cohort. **(D,E)** The forest plots for univariate **(D)** and multivariate **(E)** Cox regression analysis in validation cohort.

**FIGURE 10:**
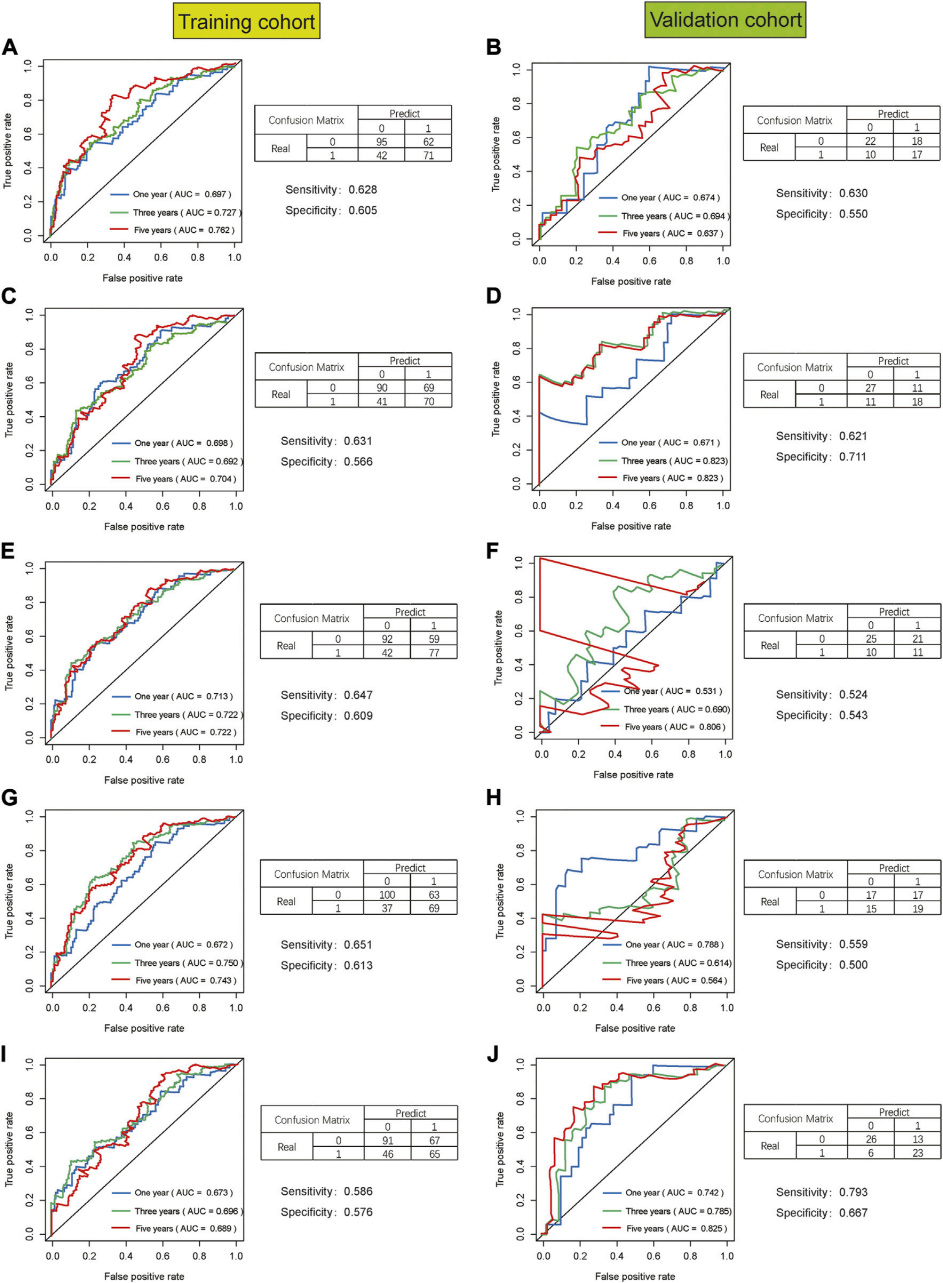
The result of 5-fold cross-validation based on seven glycolysis-related lncRNAs in STAD. AUC, confusion matrix, sensitivity and specificity were showed in each cross-validation cohort, results of training cohort on the left and validation cohort on the right in figure. **(A,B)**, **(C,D)**, **(E,F)**, **(G,H)**, **(I,J)** are the results from the first cross-validation to the fifth cross-validation.

The same Coef value was used to further verify the above results in the validation cohort (n = 170). Consistent with the results of the training cohort, the OS of high-risk patients was shorter than that of the low-risk STAD patients (*p* = 0.003) (Figure 9). The calculated AUC values of the risk model based on internal validation cohort for 1-, 3-, and 5-year survival times were 0.686, 0.699, and 0.730, respectively (Figure 8). The univariate and multivariate Cox regression analyses indicated that the risk score also was an independent predictor, affecting the prognosis of STAD (*p* < 0.001 and *p* = 0.012, respectively). In addition, the results of 5-fold cross-validation are shown in Figure 10, with similar results to the previous training dataset in most cohorts. The mean and standard deviation of the sensitivity and specificity in the cross-validated training dataset were 0.629 ± 0.012, 0.594 ± 0.010 respectively, and the sensitivity and specificity in the cross-validated training dataset were 0.625 ± 0.046, 0.594 ± 0.040 respectively.

## Discussion

The occurrence and development of tumors are closely related to abnormal cellular metabolism. Changes in energy metabolism promote the growth and proliferation of tumor cells and have been considered as emerging cancer biomarkers (Hanahan and Weinberg, 2011; Yuan et al., 2016). In 1924, Warburg discovered the abnormalities between cancerous and normal cells and showed that even under aerobic conditions, the cancer cells could maintain a higher glycolysis rate than the neighboring normal tissues, known as the “*Warburg effect*” (Warburg, 1956; Gatenby and Gillies, 2004). This phenomenon has been widely found in various tumor tissues, including GC (Liu et al., 2019), indicating that aerobic glycolysis could lead to tumor progression and poor prognosis (Luo et al., 2020; Xia et al., 2021). LncRNA is a type of non-coding RNA molecules that regulates the growth, development, and survival of cancer cells and plays an important role in multiple aspects in the initiation and progression of tumors. Therefore, the lncRNAs are considered as a novel biomarker for tumor diagnosis and prognosis (Kong et al., 2016). Many previous studies have focused on the function of specific glycolysis-related genes or prognostic signature (Liu et al., 2019; Luo et al., 2020) but there is a lack of systematic studies on glycolysis-related lncRNA as a risk signature for predicting the survival time of patients with GC. Therefore, it is necessary to construct the glycolysis-related lncRNA risk signature for the prognosis prediction in STAD, which should fulfill the deficiency of the traditional TNM stage in predicting the individualized prognosis of patients.

In this study, a total of seven glycolysis-related lncRNAs (AL353804.1, AC010719.1, TNFRSF10A-AS1, AC005586.1, AL355574.1, AC009948.1, and AL161785.1) with prognostic significance in STAD were identified using Lasso and Cox regression analyses. Among them, the two glycolysis-related LncRNAs (TNFRSF10A-AS1 and AC009948.1) have been reported in previous studies (FU et al., 2020; Wei et al., 2020). In a study by Wei et al. (Xia et al., 2021), TNFRSF10A-AS1 was identified as an autophagy-related lncRNA associated with the poor prognosis of colorectal cancer, and along with other seven autophagy-related lncRNAs, it constructed a prognostic signature for colorectal cancer, which was used to predict the prognosis in colorectal cancer patients. TNFRSF10A-AS1 was identified as a lncRNA related to glycolysis in this study. The univariate and multivariate Cox regression analyses showed that it was related to the prognosis in GC patients. These results suggested that the lncRNA TNFRSF10A-AS1 might be a common prognostic target for gastrointestinal tumors or multiple tumors and might be involved in various biological processes, such as autophagy and glycolysis. These findings also suggested that the TNFRSF10A-AS1 might participate in a variety of biological processes, affecting the prognosis of various tumors, and is worthy of further exploration in subsequent studies with good research potential. Fu et al. (FU et al., 2020) found that AC009948.1 was co-expressed with HSD11B2, which is related to the progression and prognosis of melanoma as demonstrated by mining the data of melanoma in TCGA. The GSEA analysis suggested that HSD11B2 was related to multiple cancer-related genes and pathways, including cytosolic DNA-sensing pathway, JAK_STAT signaling pathway, T-cell receptor signaling pathway, and Toll-like receptor signaling pathway. For the remaining five glycolysis-related lncRNAs (AL353804.1, AC010719.1, AC005586.1, AL355574.1, and AL161785.1), there were no published studies on their prognostic effects in cancers. Therefore, further studies are needed to explore the effects of these glycolysis-related lncRNAs on the prognosis of STAD patients.

Based on the risk signature, containing seven glycolysis-related lncRNAs, the prognosis of STAD could be significantly predicted. The AUC of 1-, 3- and 5-year OS were 0.691, 0.717, and 0.723 respectively. Similar results were obtained in the validation cohort, where the OS of the high-risk group was worser than that of the low-risk group, and the ROCs for 1-, 3- and 5-year OS were 0.686, 0.699, and 0.730 respectively. Compared with other GC-related lncRNA signatures (Wang (Wang et al., 2020) LncSig’ AUC = 0.589, Zhang (Zhang et al., 2019) LncSig’ AUC = 0.536 and Han (Han et al., 2021) LncSig’ AUC = 0.618), our glycolysis-related lncRNA signature performed better in prognosis prediction in TCGA-STAD datasets. These results indicated that this prognostic risk signature had a certain potential value for predicting prognosis in STAD patients. The risk score based on seven glycolysis-related lncRNAs could be used as independent prognostic indicators for the STAD patients through univariate and multivariate Cox regression analysis. Age and TNM stage were extracted from the training cohort to draw a concise nomogram, which was used to predict the prognosis of STAD patients. Besides, there was a good convergence between the calibration curve and standard curve. According to the results of the C-index, ROC, and calibration curve, the nomogram exhibited high discrimination and accuracy, which might become a novel potential predictive tool to provide individualized predictions in patients with GC.

Finally, although the usability of this signature in survival prediction was tested, the functions of glycolysis-related lncRNA were still unclear. Therefore, GSEA analysis was performed to explore functional differences between the high-risk and low-risk cohorts. The results of functional enrichment analysis showed that the most important pathways among these predicted glycolysis-related lncRNAs in KEGG analysis included cell adhesion, leukocyte transendothelial migration, JAK_STAT and other tumor-related classical pathways. These results could help explore and understand the mechanisms of glycolysis-related lncRNA in affecting the prognosis of STAD. Tumor invasion and metastasis are a continuous and complex process and the adhesion of tumor cells is closely related to the migration and metastasis of tumor cells. As compared to healthy tissues, tumor cells carry out glycolysis (*Warburg effect*) not only under hypoxic conditions but also in normoxic conditions, decreasing the extracellular pH (acidosis) due to increased glycolysis. As shown in a study by Hüsing et al. (Hüsing et al., 2021), after incubation at pH 6.6, the cell adhesion and migration abilities in AT1 prostate cancer cells increased by 75% and 100%, respectively, which increased lung metastases in rats. These results showed that the extracellular pH had an important effect on the migration and adhesion of tumor cells.

A previous study has shown that the JAK-STAT signal transduction pathway, widespread in humans, is related to glucose metabolism (Rastogi et al., 2007). JAK-STAT signal activation can reduce glucose metabolism by driving the expression of pyruvate dehydrogenase (PDH) kinase, which is also involved in the expression of multiple lncRNAs. The down-regulation of non-coding RNA ceruloplasmin (NRCP), identified as a highly expressed lncRNA in ovarian cancer, can lead to increased apoptosis, decreased cell proliferation, and inhibition of glycolysis (Rupaimoole et al., 2015). In addition, the lncRNA-nuclear paraspeckle assembly transcript 1 (NEAT1) is highly expressed in GC; the down-regulation of NEAT1 can inhibit the growth of GC cells and participate in the endogenous competition of miR-506/STAT3 to regulate the carbohydrate metabolism pathway in GC (Rupaimoole et al., 2015).

This study still had some limitations too. First of all, the data source of this study was single with not large sample size. Therefore, the results of the analyses might have certain deviations. Secondly, this study was a retrospective study with the inherent limitations of a retrospective study. Therefore, more prospective studies are needed to prove the predictive performance of this prognostic risk signature. Third, the validation data set of the prediction model was randomly selected as a part of the STAD samples and only internal cross-validation was carried out. We performed 5-fold cross-validation to further validate the predictive model due to lack of suitable external data for validation, but two cohorts with unsatisfactory results appeared in the validation dataset during 5-fold cross-validation (Figures: 10F and 10H). We compared the datasets in 10F and 10H with the other datasets of 10B, 10D, and 10J, respectively, and found that there were no significant differences in gender, age, tumor stage, etc. Thus, we supposed that the poor outcomes of 10F and 10H might be related to the small sample size. Therefore, further validation in other independent cohorts is necessary. Fourth, further experiments are needed to explore the potential mechanism of the lncRNAs included in the prediction model that has not been confirmed by functional experiments in the prognosis of STAD. Despite these limitations, our signature was the first prognostic risk signature based on glycolysis-related lncRNAs. In addition, the nomogram would provide clinicians with a quantitative method for predicting the survival time in STAD, which could be easily performed using Polymerase Chain Reaction (PCR) to distinguish the patients with poor prognosis from all the patients. In this way, the model might facilitate gastrointestinal oncologists to adopt a clinically individualized treatment plan. At the same time, the nomogram contained objective indicators, which could reduce differences among the observers and make the prediction of survival time more accurate.

## Conclusion

In conclusion, a seven glycolysis-related lncRNA signature has been successfully developed and verified for predicting the survival time of STAD patients. This model was used to distinguish the patients with different risks and was identified as a significant independent factor for STAD. As compared to other common prognostic factors, this prognostic signature was proved to be better. These seven glycolysis-related lncRNAs and their risk signature might act as molecular biomarkers and therapeutic targets for STAD. In addition, the nomogram with high discrimination and accuracy might provide clinicians with a novel and quantitative tool to predict the survival time in STAD patients. This model might facilitate clinicians to adopt a clinically individualized treatment plan. However, a prospective, multi-center, large-scale study is required to confirm these results.

## Data Availability

The datasets presented in this study can be found in online repositories. The names of the repository/repositories and accession number(s) can be found in the article/Supplementary Material.

## Abbreviations

GC, gastric cancer; lncRNA, long non-coding RNA; STAD, stomach adenocarcinoma; TCGA, The Cancer Genome Atlas; GSEA, Gene Set Enrichment Analysis; ROC, receiver operating characteristic; AUC, area under curve; OS, overall survival; GO, gene ontology; KEGG, Kyoto Encyclopedia of Genes and Genomes; C-index, index of concordance; CAMs, cell adhesion molecules; ECM, extracellular matrix; PDH, pyruvate dehydrogenase; NEAT1, lncRNA-nuclear paraspeckle assembly transcript 1; PCR, Polymerase Chain Reaction; SD, standard deviation; SE, standard error; HR, hazard ratio; CI, confidence intervals.
