# Supplementary figures and images for: Construction and validation of a glycolysis-related lncRNA signature for prognosis prediction in Stomach Adenocarcinoma
Source: Front Genet. 2022 Oct 14;13:794621. doi: 10.3389/fgene.2022.794621 (PMC9614251; doi:10.3389/fgene.2022.794621)

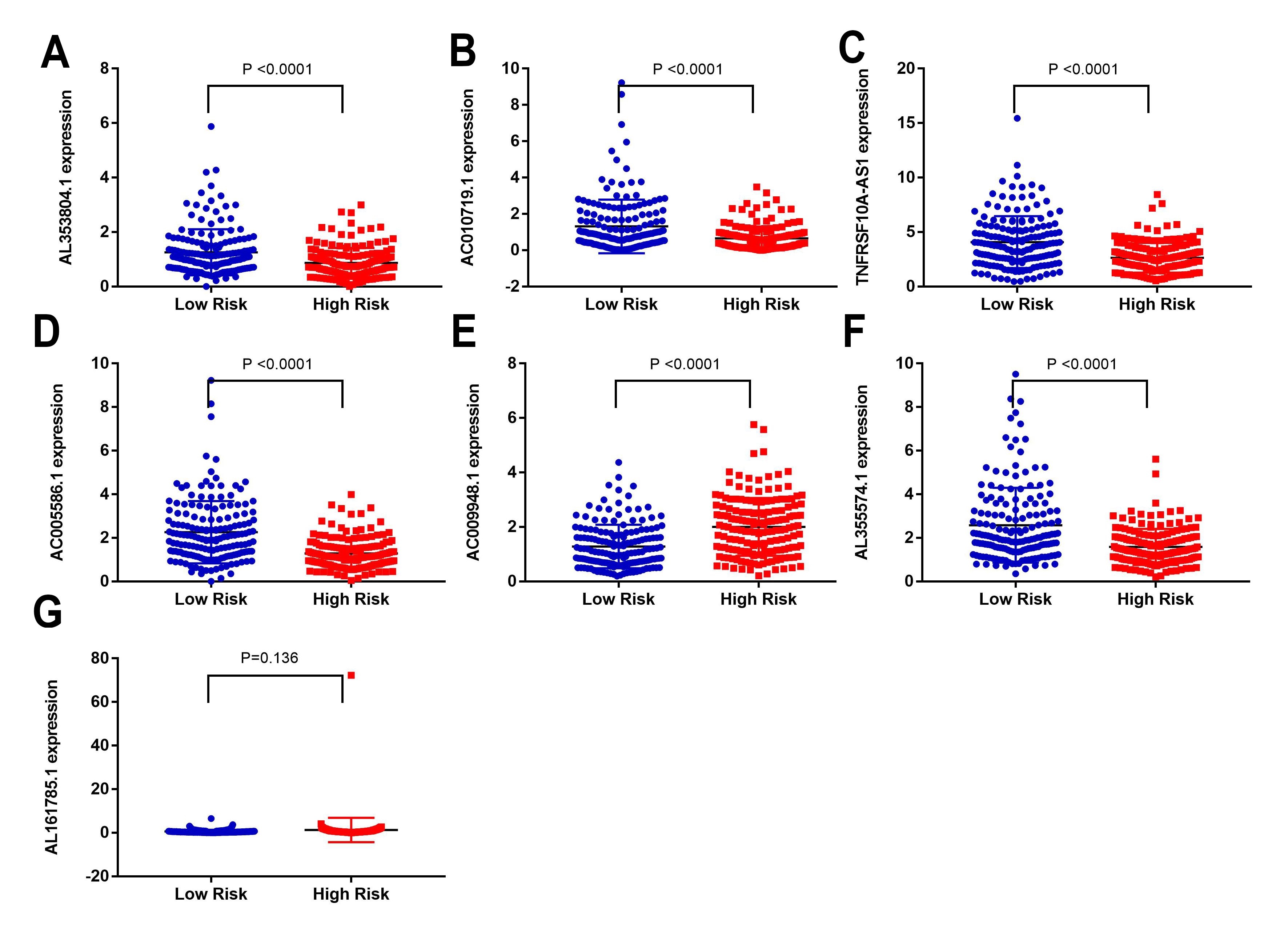

Supplement: Supplementary file 3 [file Image2.TIF]

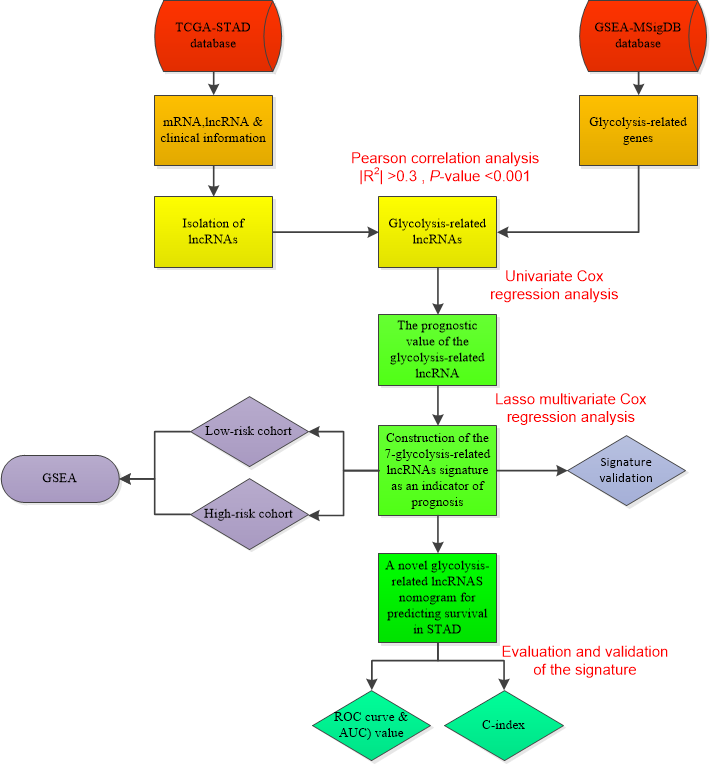

Supplement: Supplementary file 5 [file Image1.TIF]
